# Meta-analysis: COVID-19 diagnosis in chest CT—master key for radiologists

**DOI:** 10.1186/s43055-021-00457-6

**Published:** 2021-03-25

**Authors:** Soheil Hassanipour, Omid Azadbakht, Zari Dehnavi, Mohsen Shafiee, Ahmad Badeenezhad, Hossein-Ali Nikbakht, Parsa Faghani Scandarkolaei, Hassan Bostan

**Affiliations:** 1 Gastrointestinal and Liver Diseases Research Center, Guilan University of Medical Sciences, Rasht, Iran; 2 Department of Radiology Technology, Behbahan Faculty of Medical Sciences, Behbahan, Iran; 3 School of Health Management & Information Science, Iran University of Medical Science, Tehran, Iran; 4 Abadan Faculty of Medical Science, Abadan, Iran; 5 Department of Environmental Health Engineering, Paramedical School, Behbahan Faculty of Medical Sciences, Behbahan, Iran; 6 Social Determinants of Health Research Center, Health Research Institute, Babol University of Medical Sciences, Babol, Iran; 7 Behbahan Faculty of Medical Sciences, Behbahan, Iran

**Keywords:** Chest CT, Coronavirus, RT-PCR, SARS-CoV-2

## Abstract

**Background:**

COVID-19 was discovered in February in China. Due to the high prevalence of the disease, early detection and rapid isolation of patients are the vital points for controlling the outbreak. The purpose of this study was to determine the correct location of chest CT scan in the diagnosis of COVID-19.

**Main text:**

The current study is a systematic review and meta-analysis. 2959 papers were found in all national and international databases. The study has been reported based on the PRISMA checklist. All analyses were done by CMA Ver. 2 software. The statistical analysis results show that the GGO observation level in the available shape was 46% in CT scan results, and the consolidation observation level in the general form was 33% in CT scan results. Pleural effusion was 7%, and linear opacity observation level was 24% in CT scan results in the general form. The CT scan test sensitivity level was gained 94.7%, and PCR test sensitivity level was achieved as 94.8%. This level was 89% in the early stage.

**Conclusion:**

The chest CT has about 24% higher diagnostic sensitivity than the PCR test, in the early stage. GGO revealed a declining process and also indicates that GGO is an early symptom of the disease in CT scan. Linear opacity is the reason behind the initial dyspnea in coronavirus suffering patients referring to the medical centers. The extra-pulmonary lesions increase in the last stage of the disease that makes the patient’s worse.

**Supplementary Information:**

The online version contains supplementary material available at 10.1186/s43055-021-00457-6.

## Background

The large family of coronaviruses was first discovered in 1960. Currently, seven species of this family are capable of human-to-human transmission, three of which are lethal. Between 2002 and 2003, the SARS-CoV global epidemic (SARS-CoV) caused 8422 cases and killed 916 people [1]. Another strain, MERS-CoV, was first discovered in Saudi Arabia in September 2012 and eventually killed 858 people in the Middle East [2]. In December 2019, there were reports of a specific type of pneumonia in Wuhan, China. The first patients probably worked or attended one of the seafood markets in the city [3]. Since then, cases have been overgrowing, and the disease has spread to China and beyond. On February 11, 2020, the World Health Organization (WHO) announced that the official name was COVID-19 [4]. The disease primarily causes respiratory tract infection and is transmitted through respiratory droplets and contact. The incubation period is between 1 and 14 days, and the main symptoms are fever, dry cough, and fatigue; besides, nonspecific symptoms include shortness of breath, headache, and muscle pain [5]. Due to the high prevalence of the disease, early detection and rapid isolation of patients are the vital points for controlling the outbreak. Although RNA virus detection is a standard method, it still has its drawbacks and limitations. The first thing about using lab kits is that it takes a day or more to determine the test result. Also, this method can only determine the presence or absence of the disease and not judge the rate of its progression. Imaging modalities are not only useful in determining the exact location of lesions but can also help assess the extent of changes. Another is about individual people (especially medical personnel who are directly exposed to the disease); these people require regular tests and perhaps daily tests because of frequent contact. This is not possible because there is a lack of kits in the world. The next reason is the diagnostic quality of these kits. RT-PCR tests only use samples of the upper chest cavity, although in most cases, the lower chest abnormalities occur [6]. It should be noted that in addition to the above reasons, numerous studies have shown that RT-PCR kits are not highly sensitive, and in some cases give false-negative results [7–11]. Early radiological diagnosis can accelerate the planning for conservative care. Computed tomography reports of the first case of COVID-19 (a 47 years old patient) showed that the disease manifestation usually occurs in high-density, scattered shadows, mainly in the border regions of the lung. Radiology and especially computed tomography (CT) plays an essential role in the early detection, and control of this disorder. Therefore, the purpose of this study was to determine the correct location of imaging modalities in the diagnosis of COVID-19.

## Main text

### Methods

The current study is a systematic review and meta-analysis about COVID-19 diagnosis in chest CT: master key for radiologists. The study was designed and implemented in 2020. This study has been reported based on the PRISMA (Preferred Reporting Items for Systematic Reviews and Meta-Analysis) checklist.

### Search strategy

On March 17, 2020, the present study conducting researchers got to search six international databases, including MEDLINE/PubMed, ProQuest, Scopus, ISI, Embase, Cochrane library, and Google Scholar as the Gray Literature.

The selected keywords for the international databases were coronavirus, coronavirus, 2019 nCov, COVID-19, COVID 19, 2019-nCov, novel coronavirus, novel coronavirus 2019, nCov, severe acute respiratory syndrome coronavirus, Wuhan coronavirus, Wuhan seafood market pneumonia virus, and SARS-nCov2.

The collected data were entered into EndNote version X7 software, and the repeated papers were automatically removed. It is worth noting that the two researchers examined the documents separately.

### Inclusion and exclusion criteria of related studies

All studies conducted until March 17, 2020, discovered with the keywords in six databases, were included in the study. Of course, the studies whose full version was not at hand, the non-English ones, and the ones not dealing with the current topic were crossed out.

### Quality assessment

To analyze, and control the papers’ quality, the JBI checklist developed by Joanna Briggs Institute was applied, and it is used to evaluate various types of studies qualitatively. In this research, three types of lists (cross-sectional, case series, and cohort) were picked base on the inclusion study type. Each list contains different questions that are generally based on the quality of each study, as including it or not, in the final analysis, is decided.

### Screening studies

The initial search of the studies was done by two individuals (S.H, and HA.N). The screening of the studies, extracting the results, and also evaluating the papers’ quality was performed by two persons (Z.D and P.F) separately. In case of a lack of consensus, the team supervisor (O.A) announced the final comment about that paper.

### Studies’ related bias risk

Sub-group analysis and Egger tests have been employed to analyze the probability of bias in included studies.

### Statistical analysis

The heterogeneity among the studies has been checked by the Cochran test (significance level < 0.1), and its combination using *I*^2^ statistics (significance level > 50%). In the heterogeneity existence, the random-effects model was applied by the variance inversion method, and in the case of no heterogeneity, the fixed-effects model was employed.

For analyzing the data, there were seven states as GGO, consolidation, GGO, and consolidation; CP pattern; lymphadenopathy; pleural effusion; and linear opacity having the highest report in the studies were chosen. Then, the probability of observing the onset of symptoms, each of the states was analyzed based on the disease’s stage (early, progress, and advance).

To measure the two tests’ sensitivity, two tests as CT scan, and PRC were first separately employed based on the disease stage. After that, the comparison between the two trials was analyzed based on the calculated OR (odds ratio) index.

The method used in the present research, known as the calculated odds ratio (OR) index, was applied so that the readers better figure out the paper. Comprehensive Meta-Analysis (CMA) software owns the potential to combine various indices and to integrate the sample size, and the index difference is compared. According to the conclusion, the OR index can well illustrate the gap existing between the two tests. All analyses were done by CMA Ver. 2 software.

## Results

### Studies search description

**Fig. 1:**
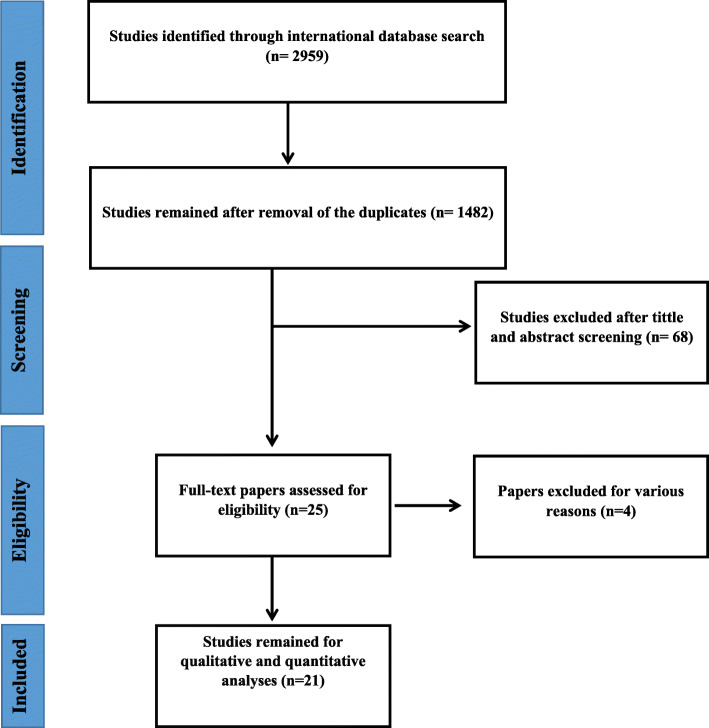
Flowchart of the included eligible studies in a systematic review

Two thousand nine hundred fifty-nine papers were found in all national and international databases that, after deleting the repeated ones, 1482 articles got into the analysis stage in terms of the title and abstract. After addressing the papers’ titles and abstracts, 68 ones got into the next step, where the full paper’s text was surveyed, and 21 reports reached the final step. It is worth stating that the included papers’ references were investigated to add the relevant articles. In the studies’ screening stages, the reviews were excluded from analysis due to various reasons encompassing irrelevant topic, irrelevant study population, and repeated results. The included studies’ flow-chart is depicted in Fig. 1.

### Included study description

The studies’ demographics are listed in Table 1. Out of 21 included studies, 17 cases were of China, two studies in Italy, and two studies in South Korea.

**Table 1:** Basic characteristics of included studies in the systematic review

Reference	Author	Location	Sample size	Type of study	Imaging modalities	Diagnosis stage	Onset of clinical sign							Rate of diagnosis in CT	Rate of diagnosis in PCR
							GGO	Consolidation	GGO & consolidation	Pleural effusion	CP pattern	Lymphadenopathy	Linear opacity		
12	Wei Xia et al	China	20	cross sectional	Chest CT	Early stage	60	50	-	-	-	-	-	100	-
13	Wei Li et al	China	5	cross sectional	Chest CT	Early stage	100	-	-	-	-	-	-	60	80
14	Xi Xu et al	China	90	cross sectional	Chest CT	Early stage	72	13	-	4	12	1	61	100	100
15	Wenjie Yang et al	China	149	cohort	Chest CT	Progress Stage	12.8	7.15	26.81	6.7	-	4.7	20.8	88.6	-
16	Yu-Huan Xu et al	China	50	cross sectional	Chest CT	Early stage	73.2	36.6	61	0.1	-	-	-	82	-
17	Heshui Shi et al	China	81	cross sectional	Chest CT	Early stage	81	-	-	5	-	14	-	100	100
17	Heshui Shi et al	China	81	cross sectional	Chest CT	Progress Stage	57	30	3	-	-	-	-	100	100
17	Heshui Shi et al	China	81	cross sectional	Chest CT	Advance Stage	33	-	-	13	-	13	-	100	100
18	Michael Chung et al	China	21	Case series	Chest CT	Early stage	57	29	-	0	19	0	14	86	-
19	Yicheng Fang et al	China	2	Case series	Chest CT	Early stage	-	-	-	-	-	-	-	100	-
19	Yicheng Fang et al	China	2	Case series	Chest CT	Progress Stage	-	-	-	-	-	-	-	100	-
10	Xie et al	China	167	cross sectional	Chest CT	Early stage	-	-	-	-	-	-	-	96	97
10	Xie et al	China	167	cross sectional	Chest CT	Progress Stage	-	-	-	-	-	-	-	98	100
8	Yicheng Fang et al	China	51	cross sectional	Chest CT	Early stage	72	-	-	-	-	-	-	98	70.5
20	Adam Bernheim et al	China	121	cross sectional	Chest CT	Early stage	44	17	-	-	0	-	0	77.68	92
20	Adam Bernheim et al	China	121	cross sectional	Chest CT	Progress Stage	88	60	-	-	20	-	20	100	92
9	Tao Ai et al	China	1014	cross sectional	Chest CT	Early stage	46	50	-	-	-	-	-	88	59
21	Harrison X. Bai et al	China	219	cross sectional	Chest CT	Early stage	91	69	64	4	5	3	51	100	-
22	Wei-cai Dai et al	China	6	Case report	Chest CT	Early stage	-	-	-	-	-	-	-	100	-
22	Wei-cai Dai et al	China	6	Case report	Chest CT	Progress Stage	-	-	-	-	-	-	-	100	-
23	Yan Li et al	China	51	cohort	Chest CT	Early stage	35.3	5.9	54.9	2	70.6	0	-	96	100
24	Shuchang Zhou et al	China	40	cross sectional	Chest CT	Early stage	47.5	37.5	-	2.5	-	-	-	100	-
24	Shuchang Zhou et al	China	22	cross sectional	Chest CT	Advance Stage	27.3	27.3	-	22.7	-	-	-	100	-
25	Wei Zhao et al	China	101	cross sectional	Chest CT	Early stage	86.1	43.6	64.4	13.9	-	1	-	92.1	-
26	Jiong Wu et al	China	80	cross sectional	Chest CT	Progress Stage	91	63	-	6	29	4	20	100	-
27	Fabrizio Albarello et al	Italy	2	Case series	Chest CT	Early stage	-	-	-	-	-	-	-	100	-
27	Fabrizio Albarello et al	Italy	2	Case series	Chest CT	Progress Stage	-	-	-	-	-	-	-	100	-
28	Soon Ho Yoon et al	Korea	9	cross sectional	chest radiography	Early stage	20	80	-	-	-	-	-	55.5	-
28	Soon Ho Yoon et al	Korea	9	cross sectional	chest CT	Early stage	35	5	50	-	10	-	-	100	-
29	Kunhua Li et al	China	83	cross sectional	Chest CT	-	97.6	63.9	-	8.4	36.1	8.4	65.1	100	-

### Results of quality assessment

The results obtained from analyzing the studies’ qualitative control based on the included reviews indicate that all reviews possess desirable quality. The total results of the studies’ qualitative assessment are covered in supplementary file 1.

### Heterogeneity-related results

Heterogeneity investigation-derived results of all analyses have been shown that in Table 2.

**Table 2:** Results of heterogeneity among included studies

Variable	Category	# of studies	Q-value	Df (Q)	I-squared	*P*-value	Selected model
GGO	Advance stage	2	0.291	1	0.0	0.590	Fixed
	Early stage	15	231.07	14	93.94	< 0.001	Random
	Progress stage	4	144.53	3	97.92	< 0.001	Random
	Overall	21	394.99	20	94.93	< 0.001	Random
Consolidation	Advance stage	1	0.0	0	0.0	0.999	Fixed
	Early stage	12	151.16	11	92.72	< 0.001	Random
	Progress stage	4	81.91	3	96.33	< 0.001	Random
	Overall	17	238.65	16	93.29	< 0.001	Random
GGO & Consolidation	Early stage	5	2.65	4	0.0	0.618	Fixed
	Progress stage	2	13.07	1	92.35	< 0.001	Random
	Overall	7	81.49	6	92.63	< 0.001	Random
CP Pattern	Early stage	5	2.65	4	0.0	0.618	Fixed
	Progress stage	2	13.07	1	92.35	< 0.001	Random
	Overall	7	81.49	6	92.63	< 0.001	Random
Lymphadenopathy	Advance stage	2	1.45	1	31.21	0.228	Fixed
	Early stage	6	18.56	5	73.06	0.002	Random
	Progress stage	2	0.11	1	0.0	0.738	Fixed
	Overall	10	31.51	9	71.44	< 0.001	Random
Pleural effusion	Advance stage	1	0.0	0	0.0	0.999	Fixed
	Early stage	7	14.76	6	59.34	0.022	Random
	Progress stage	2	0.01	1	0.0	0.893	Fixed
	Overall	10	17.98	9	50.95	0.035	Random
Linear Opacity	Early stage	4	16.53	3	81.85	0.001	Random
	Progress stage	3	0.04	2	0.0	0.978	Fixed
	Overall	7	103.26	6	94.19	< 0.001	Random
Diagnosis test (CT scan)	Advance stage	2	0.40	1	0.0	0.523	Fixed
	Early stage	21	79.62	20	74.88	< 0.001	Random
	Progress stage	6	8.97	5	44.29	0.110	Fixed
	Overall	29	89.0	28	70.52	< 0.001	Random
Diagnosis test (PCR)	Advance stage	1	0.0	0	0.0	0.999	Fixed
	Early stage	6	34.73	5	85.60	< 0.001	Random
	Progress stage	5	12.14	4	67.05	0.016	Random
	Overall	12	177.27	11	93.79	< 0.001	Random

### Results of meta-analysis

#### GGO

**Fig. 2:**
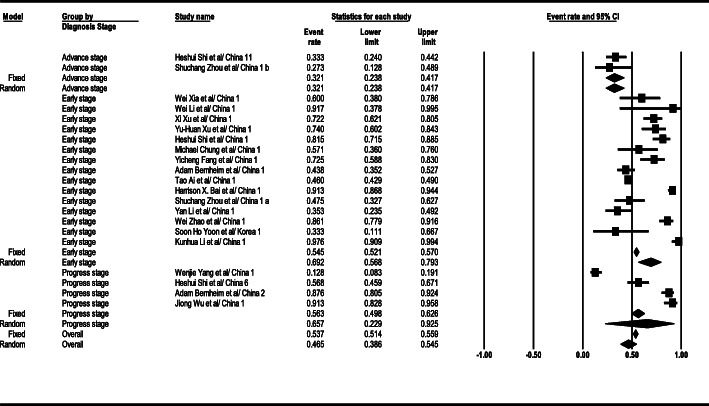
Results of GGO in CT scan of patients

As the results suggest, the GGO observation level in the general form was 46% (95% confidence intervals [CI]; 38–54) in CT scan results. This level was 69% (95% CI; 56–79) in the early stage, 65% (95% CI; 22–92) in the progress stage, and 32% (95% CI; 23–41) in the advance stage. The GGO observation findings related to total results in the patients’ CT scan are seen in Fig. 2.

#### Consolidation

**Fig. 3:**
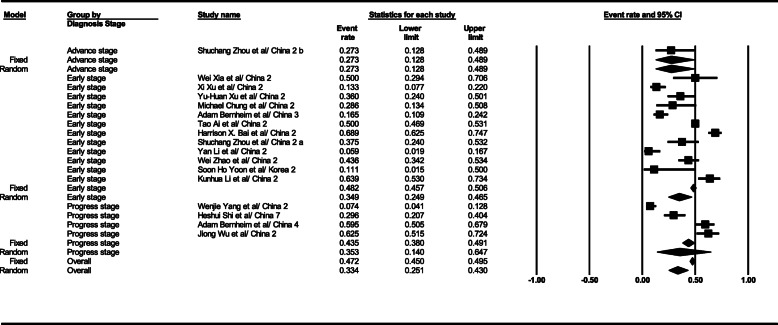
Results of GGO in CT scan of patients

The statistical analysis results show that the consolidation observation level in the general form was 33% (95% CI; 25–43) in CT scan results. This level was 34% (95% CI; 24–46) in the early stage, 35% (95% CI; 25–43) in the progress stage, and 27% (95% CI; 12–48) in the advance stage. The total results of the consolidation observation findings in the patients’ CT scan are illustrated in Fig. 3.

#### GGO and consolidation

**Fig. 4:**
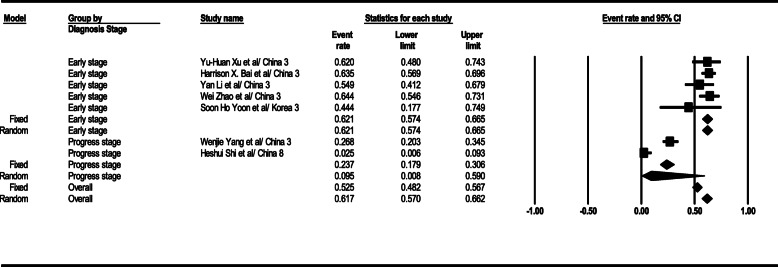
Results of GGO in CT scan of patients

According to the results, GGO, and consolidation observation level in the general form was 61% (95% CI; 57–66) in CT scan results. This level was 62% (95% CI; 57–66) in the early stage and 9% (95% CI; 0–59) in the progress stage. The total results of GGO and consolidation observation findings in the patients’ CT scan are depicted in Fig. 4.

#### Crazy paving pattern (CP pattern)

**Fig. 5:**
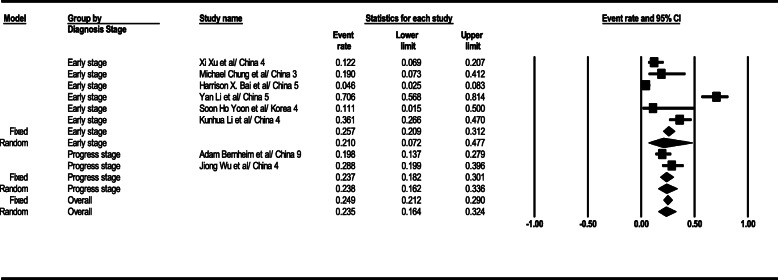
Results of GGO in CT scan of patients

Based on the results, the CP pattern observation level in the general form was 23% (95% CI; 16–32) in CT scan results. This level was 21% (95% CI; 7–47) in the early stage and 23% (95% CI; 16–33) in the progress stage. CP pattern-related total results in the ST scan of patients are shown in Fig. 5.

#### Lymphadenopathy

**Fig. 6:**
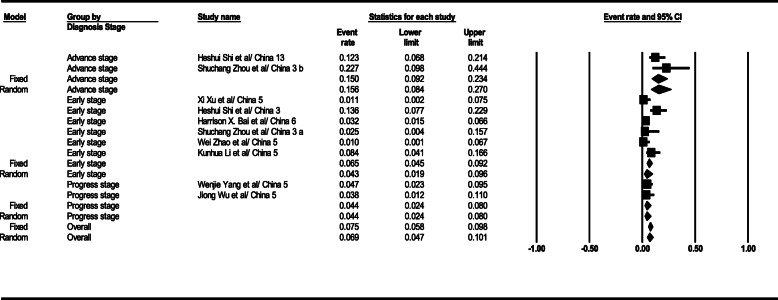
Results of GGO in CT scan of patients

The statistical analysis extracted results denoted that lymphadenopathy observation level was 6% (95% CI; 4–10) in CT scan results. This level was 4% (95% CI; 1–9) in the early stage, 4% (95% CI; 2–8) in the progress stage, and 15% (95% CI; 9–23) in the advance stage. Lymphadenopathy observation-based total results in the CT scan of patients are shown that in Fig. 6.

#### Pleural effusion

**Fig. 7:**
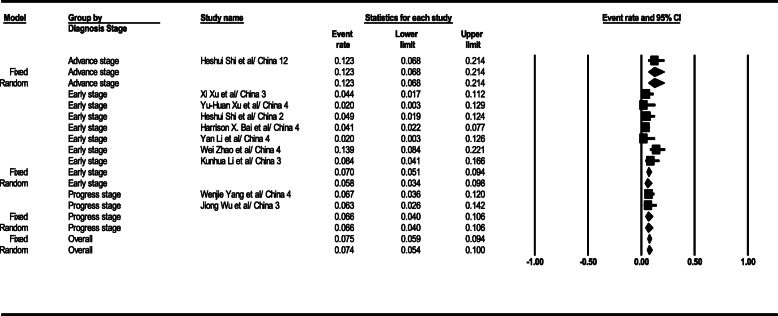
Results of GGO in CT scan of patients

The statistical analysis results indicated that pleural effusion in the general form was 7% (95% CI; 5–10) in CT scan results. This level was 5% (95% CI; 3–9) in the early stage, 6% (95% CI; 4–10) in the progress stage, and 12% (95% CI; 6–21) in the advance stage. The total results of pleural effusion observation in the patients’ CT scans are depicted in Fig. 7.

#### Linear opacity

**Fig. 8:**
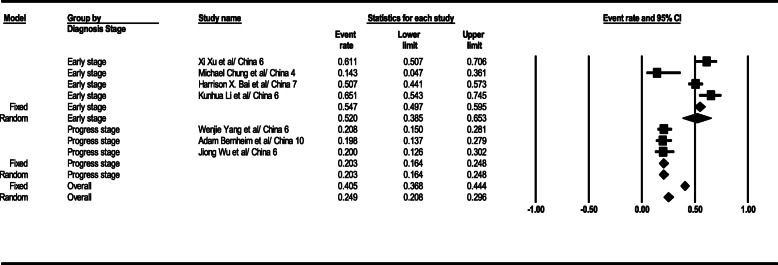
Results of GGO in CT scan of patients

Based on the results, the linear opacity observation level in the general form was 24% (95% CI; 20–29) in CT scan results. This level was 52% (95% CI; 38–65) in the early stage and 20% (95% CI; 16–24) in the progress stage. Linear opacity-related total results in the patients’ CT scans are demonstrated in Fig. 8.

#### CT scan test sensitivity

**Fig. 9:**
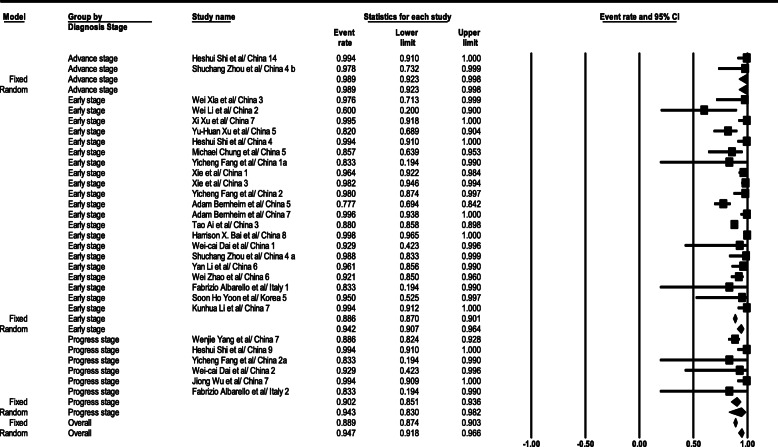
Results of GGO in CT scan of patients

As the statistical analysis results suggest, CT scan test sensitivity level in the general form was gained 94.7% (95% CI; 91.8–96.6). This level was 94.2% (95% CI; 90.7–96.4) in the early stage, 90% (95% CI; 85.1–93.6) in the progress stage, and 99% (95% CI; 92.3–99.8) in the advance stage. CT scan test sensitivity-related total results are depicted in Fig. 9.

#### PCR test sensitivity

**Fig. 10:**
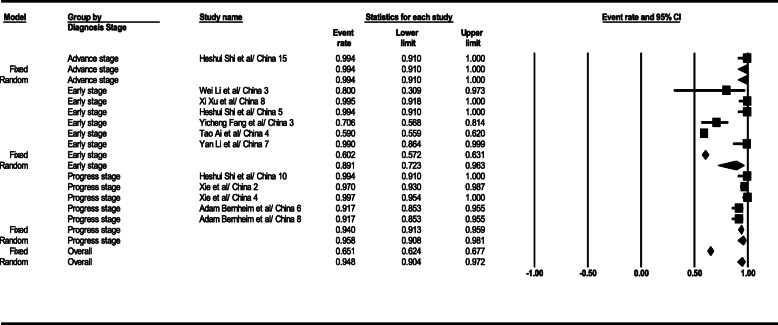
Results of GGO in CT scan of patients

According to statistical analysis results, the PCR test sensitivity level was achieved as 94.8% (95% CI; 90.4–97.2) in the general form. This level was 89% (95% CI; 72.3–96.3) in the early stage, 95.8% (95% CI; 90.8–98.1) in the progress stage, and 99.4% (95% CI; 91–100) in the advance stage. PCR test sensitivity relevant total results are given in Fig. 10.

#### Comparing CT scan and PCR tests

**Fig. 11:**
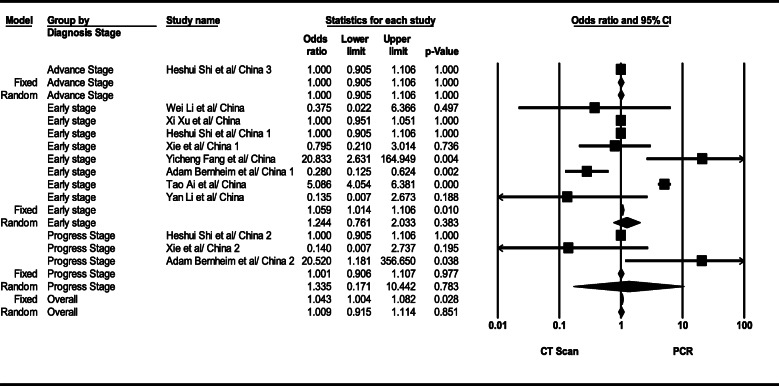
Results of GGO in CT scan of patients

As revealed by calculated OR index, generally speaking, CT scan and PCR tests did not show statistically significant (OR=1.001, 95% CI; 0.91–1.11, *p*= 0.851; *I*^2^= 94.97, *p* < 0.001), while according to data analysis, CT scan test showed approximately 24% higher sensitivity than PCR in the early stage (comparison of CT and PCR tests section, based on the data analyzed in Fig. 11), the finding which was not statistically significant (OR=1.24, 95% CI; 0.76–2.03, *p*= 0.383; *I*^2^= 94.97, *p* < 0.001). The total results of a comparison between CT scan and PCR tests are seen in Fig. 11.

#### Publication bias

**Fig. 12:**
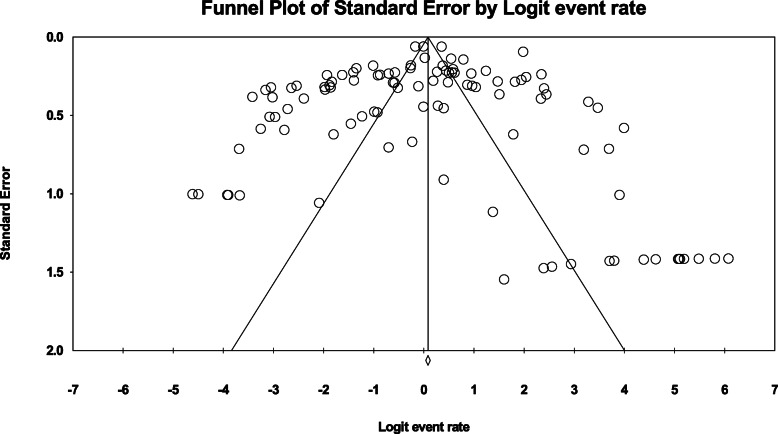
Funnel plot of standard error by Logit even rate

To investigate publication bias in the results, the Egger test and funnel plot extracted results were applied. The obtained results indicate no significant error spotted in the works (*p* = 0.747). The funnel plot of publication bias analysis is observed in Fig. 12.

## Discussion

One of the goals the current research pursues is to compare the diagnostic percent of chest CT and RT-PCR to obtain the most effective method for diagnosing COVID-19, and the findings exhibit that chest CT scan was 24% more sensitive than RT-PCR in the early stages of the disease diagnosis. Likewise, the study done by Tao Ai showed that RT-PCR plays a fundamental role in isolating and hospitalizing COVID-19 suffering patients. Still, the factors like sampling operation, sampling sources (sampling location in the respiratory tract or lung), sampling time, and the diagnostic kit’s quality impair it relative to chest CT in the early disease diagnostic stages. On the one hand, chest CT determines pulmonary engagement and progression of the disease and is less affected by human error [6–11, 30–34].

The present study-derived findings suggest that GGO outbreak level is around 69% in the early diagnostic stages of COVID-19 in chest CT. Similarly, GGO was seen in the majority of the studies on coronavirus suffering patients undergoing CT scan tests [17, 30–32, 35]. In some studies such as Li et al.’s research, only GGO was analyzed in chest CT; this issue indicates the physicians’ experimental perception of this symptom outbreak in chest CT images [13, 24, 35, 36]. Generally speaking, the abovementioned findings suggest that probably GGO is of coronavirus clinical sign in chest CT images, and this itself approves the present research [37]. It is worth mentioning that in some tasks, such as the one by Li et al., only the GGO was analyzed in chest CT. This issue indicates the physicians’ experimental perception of this symptom outbreak in chest CT images [13, 24, 35, 36]. Generally speaking, the abovementioned findings suggest that probably the GGO is of coronavirus suffering patients’ clinical symptoms in chest CT images, and this itself approves the present research [37]. Besides, as stated by the study of Xu et al., crazy paving pattern is a particular state of the GGO. Thus, it has to include a lower percentage of GGO; the issue shown by this study [35].

The studies’ analysis-derived results revealed that consolidation symptom’s incidence in chest CT images is generally 33% (a pulmonary consolidation is a region of normally compressible lung tissue that has filled with liquid instead of air), and also consolidation results from inflammatory exudation by alveoli filling, which means the virus spreads through the respiratory epithelium and leads to alveoli damage and necrotic bronchitis. It has to be stated that this finding has been investigated by Li et al. [29, 38–40]. MIX state or the simultaneous presence of consolidation, and GGO in the early stage of diagnosis has gained a high percentage (61%). This high percentage has been confirmed in several other studies [15, 21, 23, 41]. This study extracted results showing that the linear opacity is generally 24% and 52% in the early stage of diagnosis. The linear opacity indicates a decrease of air to tissue ratio due to pathogens. This is probably the reason behind the initial dyspnea in coronavirus suffering patients referring to the medical centers. The presence of the linear opacity in chest CT images of the patients has been supported in the study by Chung et al. [18]. As stated by the research of Kunhua et al., the extra-pulmonary lesions such as pleural effusion signal severe inflammation as less than 10% in the present study generally and increasing in the last stage of the disease due to the grave condition of some of the patients [29].

Comparing the different stages of the disease diagnosis in this study demonstrates that the clinical manifestations of the study did not change much (symptoms of coronavirus on chest CT imaging). However, the GGO revealed a declining process, and it is probably due to recovery over time and also indicates that the GGO is an early symptom of the disease [24, 42].

## Conclusion

To sum up, the chest CT and PCR-induced results are almost identical with no meaningful difference. Though, in the early stage of the disease diagnosis, chest CT has about 24% higher diagnostic accuracy than PCR test. In the early stage of disease diagnosis as the most critical one, the most prevalent COVID-19-induced symptoms in chest CT are GGO and Mix (GGO and consolidation). Analyzing all stages of the disease diagnosis showed that the highest incidence in chest CT images are related to GGO and Mix (GGO, and Consolidation), and the least are of pleural effusion and lymphadenopathy. Moreover, in the current research, it has been found that GGO is the early symptom of the disease and declines over time. Also, the extrapulmonary lesions such as pleural effusion signal severe inflammation in the disease advance stage due to the grave condition of some of the patients have increased.

## Supplementary Information

**Additional file 1.**

## Data Availability

The datasets used and/or analyzed during the current study available from the corresponding author on reasonable request.

## Abbreviations

CT: Computer tomography
CP Pattern: Crazy-paving pattern
PCR: Polymerase chain reaction
RT-PCR: Reverse transcription-polymerase chain reaction
