# Supplementary material for: Meta-analysis: COVID-19 diagnosis in chest CT—master key for radiologists
Source: Egypt J Radiol Nucl Med. 2021 Mar 25;52(1):86. doi: 10.1186/s43055-021-00457-6 (PMC7992511; doi:10.1186/s43055-021-00457-6)
Supplement: Supplementary file 1 — Additional file 1. [file 43055_2021_457_MOESM1_ESM.docx]

**Table1. JBI critical appraisal checklist applied for included studies**

| Cross-sectional | | | | | | | | | | | | |
| --- | --- | --- | --- | --- | --- | --- | --- | --- | --- | --- | --- | --- |
| Author Name/Year | Q1 | Q2 | Q3 | Q4 | Q5 | Q6 | Q7 | Q8 | Overall Quality | | | |
| 1-Wei Xia et al.(2020) | Yes | Yes | Yes | Yes | NA | Yes | Yes | Yes | 7 | | | |
| 2-Wei Li et al.(2020) | Yes | Yes | Yes | Yes | Yes | Yes | Yes | Yes | 8 | | | |
| 3-Xi Xu et al.(2020) | Yes | Yes | Yes | Yes | Yes | Yes | Yes | Yes | 8 | | | |
| 5-Yu Huan Xu et al.(2020) | Yes | Yes | Yes | Yes | NA | Yes | Yes | Yes | 7 | | | |
| 6-Heshui Shi et al.(2020) | Yes | Yes | Yes | Yes | Yes | Yes | Yes | Yes | 8 | | | |
| 9-Xie et al.(2020) | Yes | Yes | Yes | Yes | Yes | Yes | Yes | Yes | 8 | | | |
| 10-Yicheng Fang et al.(2020) | Yes | Yes | Yes | Yes | Yes | Yes | Yes | Yes | 8 | | | |
| 11-Adam Bernheim et al.(2020) | Yes | Yes | Yes | Yes | Yes | Yes | Yes | Yes | 8 | | | |
| 12-Tao Ai et al.(2020) | Yes | Yes | Yes | Yes | Yes | Yes | Yes | Yes | 8 | | | |
| 13-Harrison X Bai et al.(2020) | Yes | Yes | Yes | Yes | Yes | Yes | Yes | Yes | 8 | | | |
| 16-Shushang Zhou et al.(2020) | Yes | Yes | Yes | Yes | Yes | Yes | Yes | Yes | 8 | | | |
| 17-Wei Zhao et al.(2020) | Yes | Yes | Yes | Yes | NA | Yes | Yes | Yes | 7 | | | |
| 18-Jiong Wu et al.(2020) | Yes | Yes | Yes | Yes | NA | Yes | Yes | Yes | 7 | | | |
| 20-Soon Ho Yoon et al.(2020) | Yes | Yes | Yes | Yes | No | Yes | Yes | Yes | 7 | | | |
| 21-Kunbua Li et al.(2020) | Yes | Yes | Yes | Yes | No | Yes | Yes | Yes | 7 | | | |
| Case-series | | | | | | | | | | | | |
| Author Name/Year | Q1 | Q2 | Q3 | Q4 | Q5 | Q6 | Q7 | Q8 | Q9 | Q10 | Overall Quality | |
| 7-Michael Chung et al.(2020) | Yes | Yes | Yes | Yes | Yes | Yes | Yes | Yes | Yes | Yes | 10 | |
| 8-Yicheng Fang et al.(2020) | Yes | Yes | Yes | Yes | Yes | Yes | Yes | Yes | Yes | Yes | 10 | |
| 19-Fabrizio Albarello et al.(2020) | Yes | Yes | Yes | Yes | Yes | Yes | Yes | Yes | Yes | Yes | 10 | |
| Cohort | | | | | | | | | | | | |
|  | Q1 | Q2 | Q3 | Q4 | Q5 | Q6 | Q7 | Q8 | Q9 | Q10 | Q11 | Overall Quality |
| 4-Wenjie Yang et al.(2020) | Yes | Yes | Yes | Yes | Yes | Yes | Yes | Yes | Yes | Yes | Yes | 11 |
| 15-Yan Li et al.(2020) | Yes | Yes | Yes | Yes | Yes | Yes | Yes | Yes | Yes | Yes | Yes | 11 |
| **Cross-sectional questions:**  Q1. Were the criteria for inclusion in the sample clearly defined?  Q2. Were the study subjects and the setting described in detail??  Q3. Was exposure measured in a valid and reliable way?  Q4. Were objective, standard criteria used for measurement of the condition?  Q5. Were confounding factors identified?  Q6. Were strategies to deal with confounding factors stated?  Q7. Were the outcomes measured in a valid and reliable way?  Q8. Was appropriate statistical analysis used?  **Case Series design questions:**  Q1. Were there clear criteria for inclusion in the case series?  Q2. Was the condition measured in a standard, reliable way for all participants included in the case series?  Q3. Were valid methods used for identification of the condition for all participants included in the case series?  Q4. Did the case series have consecutive inclusion of participants?  Q5. Did the case series have complete inclusion of participants?  Q6. Was there clear reporting of the demographics of the participants in the study?  Q7. Was there clear reporting of clinical information of the participants?  Q8. Were the outcomes or follow up results of cases clearly reported?  Q9. Was there clear reporting of the presenting site(s)/clinic(s) demographic information?  Q10. Was statistical analysis appropriate?  **Cohort design questions:**  Q1: Were the two groups similar and recruited from the same population?  Q2. Were the exposures measured similarly to assign people to both exposed and unexposed groups?  Q3. Was the exposure measured in a valid and reliable way?  Q4. Were confounding factors identified?  Q5. Were strategies to deal with confounding factors stated?  Q6. Were the groups/participants free of the outcome at the start of the study (or at the moment of exposure)?  Q7. Were the outcomes measured in a valid and reliable way?  Q8. Was the follow up time reported and sufficient to be long enough for outcomes to occur?  Q9. Was follow up complete, and if not, were the reasons to loss to follow up described and explored?  Q10. Were strategies to address incomplete follow up utilized?  Q11. Was appropriate statistical analysis used? | | | | | | | | | | | | |
